# Implications of Tamarkoz on stress, emotion, spirituality and heart rate

**DOI:** 10.1038/s41598-021-93470-8

**Published:** 2021-07-08

**Authors:** Nasim Bahadorani, Jerry W. Lee, Leslie R. Martin

**Affiliations:** 1 Center for Nutrition, Healthy Lifestyle, and Disease Prevention, Loma Linda University|School of Public Health, 24951 North Circle Drive, Nichol Hall 1511, Loma Linda, CA 92350 USA; 2 Center for Nutrition, Healthy Lifestyle, and Disease Prevention, Loma Linda University|School of Public Health, 24951 North Circle Drive, Nichol Hall 1313, Loma Linda, CA 92350 USA; 3 Department of Psychology, La Sierra University, 4500 Riverwalk Parkway, Riverside, CA 92505 USA; 4 Present Address: P.O. Box 72424, Davis, CA 95617 USA 72424,

**Keywords:** Lifestyle modification, Preventive medicine, Public health, Quality of life, Human behaviour

## Abstract

Perceived stress among university students is a prevalent health issue directly correlated with poor academic performance, poor sleep quality, hopelessness, compromised physical and mental health, high risk of substance abuse, and suicidal ideation. Tamarkoz, a Sufi meditation, may reduce the impact of stressors to prevent illness among students. Tamarkoz is the art of self-knowledge through concentration and meditation. It is a method of concentration that can be applied to any task. The method is said to discipline the mind, body, and emotions to avoid unintended distractions. Therefore, it can be used in daily life activities, such as studying, eating, driving, de-stressing or in Sufism, seeking self-knowledge. This study was an 18-week quasi-experimental design with pre-intervention, post-intervention and follow-up assessments in the experimental group, a wait-list control, and a third group that utilized the campus health center’s stress management resources. Participants, university students, had no prior exposure to Tamarkoz, and there were no statistically significant differences among groups on baseline measurements. Using a generalized linear mixed model, significant increases in positive emotions and daily spiritual experiences, and reductions in perceived stress and heart rate were found in the experimental group compared to the other two groups. Tamarkoz seems to show some advantages over the usual stress management resources offered by a student health center.

Trial registration: ClinicalTrials.gov Protocol Registration Date: (03/04/2018); ClinicalTrials.gov ID: NCT03489148.

## Introduction

Perceived stress is a prevalent health concern^1^ and a neglected global public health issue which has significant negative influence on students’ quality of life^2^ as it is directly correlated with drop-out^3^, poor sleep quality^4^, somatic pain^5^, hopelessness^6^, compromised mental and physical health^2,7^, high risk of substance abuse^8^, depression^9^ and suicidal ideation^6^. Perceived stress is psychological and is induced when a situation or stimulus is assessed as threatening, irrespective of the actual threat value^1^. Students' stress stems from a variety of pressures including social, financial, personal, and academic challenges^10,11^. Non-medical use of prescription stimulants such as Adderall, Ritalin, and Vyvanse are common as they attempt to enhance their academic performance^12^.

Perceived stress is directly correlated with depression- the number of college students diagnosed with depression is increasing and the prevalence of anxiety is high^13^. Demands for counseling services are increasing^14,15^, but campuses have limited resources to meet these needs^14^. It is therefore pragmatic to examine alternative methods for stress management^16^ and provide students with opportunities to develop skills for reducing the physiological and psychological effects of academic stress^13,17^.

The physiological response to stressors, allostasis, is the body's attempt to maintain homeostasis by releasing glucocorticoids, epinephrine, and other mediators^18^. Over time, this allostatic load takes a toll on the body, but things can be done to help mitigate the negative effects of the stress response and to minimize over-reactivity^18^. Recovery takes place when the stress systems are no longer activated (i.e., relaxation) and this recovery is vital to maintaining physical and mental health, and well-being^18,19^. A relaxation response can protect against stress-related conditions, and activities such as deep breathing, prayer, and meditation evoke this response at the level of the central nervous system^20–22^.

The central nervous system regulates cardiac and respiratory rhythms, which are functionally related^23^. Respiration can modulate heart rate which, in turn, influences the sympathetic and parasympathetic response of autonomic nervous^24^. The phenomenon known as cardiorespiratory synchronization is an interaction between the respiratory system and the human heart^25^. Research shows that meditation enhances cardiorespiratory synchronization^24,26^ compared to spontaneous breathing and normal relaxation^25,27^, and strong synchronization indicates a healthier physiological state^25,27^.

Cortical activity can also affect cardiorespiratory synchronization. For example, an emotional or cognitive task may arouse sympathetic activity and change breathing rhythm which then increases heart rate^28^. In contrast, meditation and mind–body interventions with slow breathing can prompt changes in brain activity which work to inhibit sympathetic and limbic responses, and promote the parasympathetic function^24^. During a prominent parasympathetic activity, there is an increase in alpha waves and functional connectivity in the brain^24^. The higher levels of cardiovascular synchronization present during meditation lead to hyperpolarization and inhibition of cortical neurons. This results in a homeostatic modulation of the autonomic nervous system and brain activity^24^. As such, the effects of meditation on homeostasis in the body are more substantial than relaxation alone. Meditation, although relaxing, is a process that inhibits irrelevant thought to selectively focus on a specific target^29^. The alpha activity measured from EEG during this meditative resting state may be considered thalamocortical and cortical oscillations which have been associated with higher brain functioning and performance^29^.

There are many different types of meditation such as mindfulness-based stress reduction (MBSR), Transcendental Meditation (TM), and the Tamarkoz method of meditation. The Tamarkoz method is defined as the art of self-knowledge through concentration and meditation^30^. It is unique to M.T.O. Shahmaghsoudi, the School of Islamic Sufism. The practice of Tamarkoz includes heart-focused meditation, movement balancing meditation, a state of deep relaxation, concentration and visualization, mind relaxation exercises and deep breathing techniques^30^.

The mind relaxation exercises coupled with deep breathing in the Tamarkoz method are said to control the practitioner’s habitual interfering thought patterns, control emotional reactivity, consciously slow down brain waves, and prepare the practitioner physically and mentally for concentration^31^. During the state of deep relaxation, the participant releases muscular and nervous tension and dissipates mental and physical fatigue^32^. As cellular energetic resources are renewed, the participant becomes open to connecting with a deeper dimension of one’s being^32^. Visualization or imagery in the Tamarkoz method serves to replace negative thought patterns with positive healing images that could be based on the participant’s past experiences^33^. All five senses are used during this exercise which also provides neurological and sensory training for the participant to develop laser focus on a single point^33^. The visualization exercises stimulate hope, creativity, motivation and vitality^33^.

There is substantial evidence in scientific literature that individual techniques similar to those used in Tamarkoz, including deep breathing^17^, guided visualization^34^, and slow physical movement^35^ have physiological effects resulting in improved health outcomes. Benefits such as increase in multiple facets of mindfulness, which have important mental and behavioral health implications associated with improved mood and reductions in perceived stress, appear greater with meditative movements than sitting meditation^36^. MBSR has been shown to significantly decrease perceived stress^37^, distractive ruminative thoughts and behaviors^2,22^, depression and anxiety^2^, and significantly increase positive moods^22^, self-compassion, and hope^37^. TM has been shown to significantly improve distress, anxiety, and depression^38^. The benefits are not limited to well-being, but also extend to improve cognitive functioning (e.g., knowledge retention in college students) by facilitating mindful control over one’s attention, and directing sustained attention on a chosen thought or object^39–41^. While the positive health effects of some forms of meditation are well-established in scientific research, to our knowledge there has been little published research in scientific peer-reviewed journals on the Tamarkoz method.

The various meditation practices have similar effects on balancing the nervous system and inducing a relaxation response that ultimately promotes health with stress reduction^16,17,19,36^. However, they differ in the manner of practice, the traditions from which they stem, the ways they are learned, their physiological effects, and the purpose for the practices (see Table 1). For example, MBSR, and TM are secularized, whereas Tamarkoz is a spiritual practice. Research has demonstrated significant positive correlations between spirituality and health outcomes, and studies show spirituality may improve health and reduce stress^42^.

**Table 1:** Comparison of meditation techniques.

Method	Mindfulness	Relaxation response	Transcendental meditation	Tamarkoz
Brain's Default Mode Network (DMN)	Deactivates DMN		Activates DMN	
Training Style	8-week course on MBSR developed by Jon Kabat-Zinn. Or through various websites, and books	A variety of activities can elicit this physiological state of relaxation	Requires a certified TM instructor with a standardized technique	Taught by instructors who are approved by the Sufi Master of M.T.O. Shahmaghsoudi
Focus of Practitioner	The mind and embodied experience including scanning bodily sensations in an orderly manner	The mind	The mind	The heart
Brain Wave Patterns	Theta waves	–	Alpha waves	Alpha waves
Practice Components	Training one's mind to be in the present moment in any daily activity. Passive attention to one's breathing, sensations, and thoughts without judgement	One way is repetition of a word, phrase or prayer while sitting at ease without movement in a comfortable position and all muscles relaxed. Breath slowly. For 10–20 min	Described as an effortless, simple, natural technique with use of a manta to let the mind settle down naturally and ultimately to transcend thought. Practice is 20 min/day	Deep breathing, mind relaxation, movement balancing called Movazeneh, guided visualization, and heart Tamarkoz
Goal	To have one's thoughts be on the present moment. Attempt is not on quieting the mind, but sustaining focus and emotional release to change behavioral habits and thinking on a foundational level to improve wellbeing^45, 84^	Relaxation by eliciting the parasympathetic response	State of relaxed awareness or transcendence of thoughts. Allows the mind to settle to quiet levels of thought	To have no thoughts. Focus on self-discovery. Connecting with electromagnetic centers in body
Spiritual or Religious practice	No	No	No	Yes
Applicable to anyone irrespective of religious affiliation or lack thereof?	Yes	Yes	Yes	Yes
Origin	Buddhist tradition popularized and secularized by Jon Kabat-Zinn with Mindfulness Based Stress Reduction	A simpler and more secularized version of TM coined as term by Herbert Benson	Vedic tradition and secularized by Maharishi Mahesh Yogi who brought it to India in 1955, and then to the US in 1959^42^	A 1400-year practice of Sufism. In its current form developed by the Sufi Master Professor Nader Angha

Spirituality has been incorporated into treatment interventions with mindfulness-based therapies which focus on awareness of an experience and its consequent effects^43^. Although MBSR is a secularized behavioral medicine program, it has roots in Buddhist meditative spiritual practice^44^. Participation in a MBSR program has been shown to significantly increase both mindfulness and spirituality scores and also to reduce psychological distress^44^. Just as a spiritual aspect is sometimes integrated with mindfulness-based interventions, research has also demonstrated that mindfulness-based interventions can enhance spiritualty^44^. This increase in spirituality is especially pronounced with interventions that have a heavy focus on spirituality, such as with some substance-use recovery programs^45^.

Practitioners of spiritual meditation have demonstrated better coping, increased pain tolerance, better mental health, more positive moods and less anxiety than secular meditation practitioners^46^. Spiritual meditation has also been shown to reduce stress reactivity through feelings of spiritual connection, feelings of peacefulness and calmness, and increased self-efficacy^46^.

Although elements of spirituality (e.g., spiritual well-being, spiritual dimension, spiritual awareness) are found in research literature, it is argued that the essence of spirituality cannot be adequately defined with language^47^. Nevertheless, some researchers emphasize the importance of detailed definitions for research purposes despite the majority’s conclusion that there is not a unified definition of spirituality^42,47^. Some define spirituality as intrinsic beliefs and personal values that guide daily living^47^, others define it as the core of one’s existence and suggest that it gives significance to people's lives, and some consider spirituality and religiosity to be interchangeable^42^.

Health scientists see spirituality as distinct from religiosity^47^. Religion may be defined from a health science perspective as a multidimensional construct and an organized system of beliefs, practices and symbols designed to foster closeness to God or the sacred, whereas spirituality has been defined as the connection to that which is sacred^48^. These definitions overlap, but the distinction between religion as adherence to particular practices and beliefs may sometimes conflict with forms of spirituality^49^. Research shows that both religiosity and spirituality can help people deal with adversity, illnesses, and other stressful situations^48^. However, although religiosity has sometimes been shown to be an effective coping mechanism, the type of religious coping is a critical component in psychological outcomes^49^. Positive religious coping is correlated with positive outcomes while negative religious coping is correlated with negative outcomes^42^.

A belief in God may be another factor that some use to differentiate between being spiritual and religious. Atheists and agnostics do not believe in a God and may be doubtful of organized religion^50^ but may be spiritual or experience associated feelings such as connectedness, harmony, love, humility, emotional stability, compassion, and more^51^. Although there are many different definitions of spirituality^52^, it is commonly viewed as being developed through prayer and meditation^53^. Religion may provide guidelines through which spirituality may be developed. For example, it may be viewed as a training system through which individuals can learn to discover divine representations in themselves^53^.

In the discipline of Islamic Sufism, from which the Tamarkoz method of meditation derives, the goal is to achieve knowledge of one’s unlimited true self called the “I,” which is said to be located in each person’s physical heart^53^ and which is referred to as the “Source of Life,” in the heart by the great Sufi Master Professor Sadegh Angha^54^. Thus, meditation and concentration on the heart is important in Sufi teachings and practices, as it is said to be the gateway to the spiritual dimension of a human being^53^. The spiritual dimension is one’s connection to all existence which is the discovery of the unlimited true self. This is said to occur when you “Gather all your energies and concentrate them on the source of life in your heart for your findings to become imperishable, so that you will live in balance and tranquility and know eternity”^54^. This form of concentration in the heart enables one’s connection to all existence and the ability to find tranquility and balance. Tamarkoz, taught by instructors who have been approved by Sufi Master Professor Nader Angha, is one approach to facilitating this sense of tranquility and balance utilized by the M.T.O. Shahmaghsoudi, School of Islamic Sufism.

Individuals do not have to be followers of the Sufi faith to participate in the practice. Tamarkoz is said to create a state of equilibrium, balance and harmony for an individual^53^. Harmony is said to be achieved when the entire being is present and not focused on thoughts, emotions, the senses, or any other factors external to oneself^53^. Thoughts, emotions and senses are not considered to be part of the true self in Sufism, because these are all subject to change and anything that changes is not considered to be truth^53^. Furthermore, the senses are limited in their perceptions.

For example a typical human eye is sensitive to wavelengths between 380 and 750 nm on the electromagnetic spectrum^55^. A Tamarkoz practitioner is said to meditate within the heart to become sensitive to electromagnetic waves that do not reach the threshold of action potentials^56^. Rather, M.T.O. Shahmaghsoudi teaches that the heart is capable of receiving these subtle electromagnetic waves which are then transmitted to the brain for processing and amplification so that they can reach threshold for action potentials, provided that the brain dismisses other potentially interfering stimuli^56^. This is one of the many ways that Tamarkoz is different from mindfulness or other forms of meditation. The result of such a connection between the heart and brain is the discovery of “I,” which is equated with achieving oneness with existence. The components of the practice are used to quiet the mind so one’s concentration and focus is directed on the heart.

Christian literature also refers to the heart. For example, Prayer of the Heart (PH) is the central contemplative practice of the Eastern Christian traditions Apophthegmata and Hesychasm^57^. Some consider the concept of the heart in this prayer to be metaphorical and symbolic, however phenomenological analysts explain how Prayer of the Heart are actual embodied psychosomatic experiences^57,58^. The PH experience is described as an egological awareness of the body-self in the chest area associated with sensations, emotions, feelings, and verbal thoughts^57^, which is different from Tamarkoz meditation. According to Sufi teachings, the human being is not limited to the body and one’s true self or “I” is not a psychological system developed in the mind. In Sufism, the body is considered a vehicle or a tool by which one can develop one’s inner connection to the unlimited within the human heart. The brain is considered part of the body and thus, self-identity is not achieved from the mind or consciousness. Judgments, perceptions and emotions are subject to change and so they are also not considered part of the stable, true self. The practices of Sufism, such as Tamarkoz, are methods of connecting to one’s true self within the human heart which is said to be boundless and not limited to the senses, mind or the physical body. As taught in Sufism, this true self called the “I,” is stable, never subject to change and resilient. No matter any changes or challenges that occur in one’s external environment including the body, one always remains stable, balanced, and resilient because one is deeply rooted within the being and connected to a stable true self^53^.

Along with meditation in the heart, another component of Tamarkoz is Movazeneh, which is slow movement and balancing meditation. It gathers the practitioner’s attention and focus to a single point that expands to the entire body through concentrated movements, which allows one to experience the present moment^59^. Table 2 provides a comparison between Movazeneh and other movement meditations.

**Table 2:** Comparison of meditation approaches to movements.

Movement meditation	Tai-Chi	Qi﻿gong	Yoga	Movazeneh
Purpose and focus of the practice	Guided by the mind and to circulate and balance the Yin and Yang aspects of chi also known as qi (vital energy)	To improve physical fitness and enhance overall well-being through flow of (qi) energy in the body^85^. Regulates flow of qi in the body. Subdivided into internal or external qi-gong. Internal qi-gong is self-directed and involves movements, and control of breathing patterns. External qi-gong refers to a practitioner who directs energy towards someone to improve the flow of their qi or treat a condition^85^	The purpose of ancient yoga practices is to achieve self-awareness and spiritual attunement^61^ by clearing blockages in the energy channels of the body. Focus is on the chakras along the human spinal column	Defined as the art of self-knowledge through concentration and meditation. Aims to bring the human being’s emotional, physical, mental and spiritual aspects into a state of balance by activating and harmonizing the body’s 13 electromagnetic centers
Origin of practice	Draws on Taoist principles. Tai Chi Chuan, the original form, traces back China almost 400 years in the Chenjiagou Village as a martial art. Many variations and sub-styles have been developed since then	Ancient martial art originates from China	Rooted in Indian philosophy. Began as a spiritual practice thousands of years ago. Today is popular for promoting physical health and wellbeing	M.T.O. Shahmaghshoudi, School of Islamic Sufism that traces back 1400 years. Required to be approved by the Sufi Master of M.T.O. Shahmaghsoudi to teach this practice
Practice components	Relaxed circular movements, postural alignments, and shifting of weight^40^	Term for health exercises in traditional Chinese medicine to improve flexibility and relaxation of the body	Although ancient yoga includes other elements, the yoga popularly practiced in the United States includes asanas (physical postures), pranayama (breathing techniques), and dyana (meditation)	Balancing movements and stretching. Movements and postures may include sitting, standing, or laying on the floor while harmonizing inhalations and exhalations with the movements and postures
Used for self-defense or combat	Yes, if practiced quickly	Yes	No	No

Movazeneh is said to direct the practitioner’s mind from a scattered to a collective state as the movement and postures stimulate the relevant nerve fibers of a specific body area which generates action potentials to the brain to increase related interferences, and disciplines the practitioner to avoid unintended abortive interferences and thereby induce higher levels of concentration^56^.

Movazeneh is also said to balance and activate the electromagnetic system in the human body^59^, whereas the practices of Tai-Chi, Qigong, and yoga focus on encouraging the flow of energy called qi or chi in traditional Chinese medicine, and Prana in Hindu philosophy. Each of these different practices focuses on highly organized energy systems in the human body. Non-Western traditional medicine and Eastern medicine developed healing practices based on observations of the entire body system of the living human being and relationships to specific symptoms^60,61^, in part because dissection of cadavers was prohibited^61^. They discovered highly organized energy systems that maintain overall wellbeing, but the energy systems cannot be directly observable by scientific measurements^61^.

The energy system of Eastern traditional medicine is known to have channels throughout the body through which energy flows. For example, according to traditional Chinese acupuncture theory, meridians are channels in the body through which qi flows^62,63^. Qi is a vital life energy in traditional Chinese medicine^63^, Tai-Chi, and Qigong. There are believed to be twelve^63^ or forteen^62^ main meridian channels longitudinally distributed on the body and named for major organs presumed to be affected by each meridian (e.g., heart, lung, gall bladder), in addition to smaller channels that branch from the main channels and reach deeper inside the body^62^. According to traditional Chinese medicine, illness or disease occurs when there is disruption in the flow of qi also known as chi; thus practices such as Tai-Chi, Qigong, and acupressure are devised to restore the flow. Chi functions according to the same concept as Kundalini energy or prana. The ancient practice of classical yoga also serves to improve the flow of prana or Kundalini to promote health and wellbeing. According to Indian philosophy, prana is the universal life force, and Kundalini is a dormant form of this universal energy that is said to be in the base of the spine^64^. Through yoga movements and breathing, the Kundalini energy becomes “awakened” and moves upwards in the spine through channels called chakras to the crown of the head^64,66,67^ enabling spiritual awareness^64^.

According to ancient Indian Ayurvedic medicine, there are seven main chakras in the human body which are considered to be energy vortices that increase in energy level along the spine and head^67^. Many of these chakras have the same name as the locations of the electromagnetic centers in the human body, but these chakras are located along the spine at the level of the organs, whereas the electromagnetic centers are located in the named organ.

According to the teachings of M.T.O. Shahmaghsoudi there are thirteen important electromagnetic centers in the human body with the main center located in the heart^53,68^, specifically in the Crista Terminalis^53^. The other important centers are located in the cerebral cortex, crown of the head (anterior fontanel and gray layer), third eye, third ventricle in the brain, brain stem, throat, thymus, three other nodes in the heart, solar plexus and the coccyx^66^. Based on the teachings, spirituality is experienced with activation and balance of these electromagnetic centers in the human body^53^.

Although science has not yet clearly defined these electromagnetic centers in the human body, the electrical properties of the heart can be measured with the electrocardiograph, the activities of the brain can be measured with electroencephalogram, and the electromyogram can be used to measure electrical activity in muscles. Additionally, scientists in the field of biomagnetism have made discoveries regarding measurement of the magnetic field of the human body and its specific organs, including the heart and brain^65,69,70^. Although the electrical and magnetic properties of the body are measurable, the electromagnetic centers have yet to be understood by science^53^.

As there is a focus on activating, balancing, and harmonizing the electromagnetic energy centers in the body during the Tamarkoz method, deep breathing exercises during the practice are said to fuel these energy centers. The oxygen provided in deep breathing along with concentration and the visualization of a particular anatomy, increases energy production and the desired electromagnetic waves in the area^56^. Among other benefits, deep breathing techniques control the depth, rhythm and the duration of the breath while also increasing respiratory capacity for maximal oxygenation, and stimulation of the immune system^71^. Furthermore, it is said the breathing techniques help to cleanse the cells and nerve channels for advanced control of subtle energies^71^. The explanation of how deep breathing cleanses the immune system and the central nervous system as well as how it enhances receptivity and amplification of electromagnetic waves is written with meticulous detail in the scientific book entitled “*Expansion and Contraction within Being (Dahm),”* by His Holiness Professor Nader Angha, Sufi Master of M.T.O. Shahmaghsoudi, School of Islamic Sufism.

Although there may not yet be a device that measures the level of activation for electromagnetic centers in the body, there are other measurable, physiological benefits to movement meditation and balancing^72^. Physical movement increases blood circulation which increases oxygen to tissues and enhances lymph flow in lymphatic vessels which improves immune function^73^. Other benefits of movement meditation include improved balance, flexibility, stability and strength^72^.

One of the places Tamarkoz is taught is at the University of California, Berkeley through the student-run democratic education program*.* The course is enrolled to capacity every semester with diverse students from different majors and has a waitlist of well over 100 people. The course provides a rich opportunity for studying this spiritually-based meditation as a potential health-promotion and disease-prevention tool. This was the aim of the present study, with the specific hypotheses that participants in the Tamarkoz class would have (a) reduced perceived stress, (b) increased positive emotions, (c) more frequent daily spiritual experiences, (d) decreased blood pressure, and (e) decreased heart rate compared to two control groups.

## Methods

### Design

This study was a three-group quasi-experiment. The three groups were: (a) a Tamarkoz group, (b) a stress management (standard practice) control group, and (c) a Tamarkoz waitlist control group. Participants self-selected into the Tamarkoz group or the stress management group. All groups were measured at three points in time: (a) at baseline near the beginning of the fall semester, (b) approximately 12 weeks after baseline, and (c) approximately 18 weeks after baseline**.**

### Participants

Inclusion criteria were that participants be University of California, Berkeley students between the ages of 18–30 years; not work third shifts; and not have diabetes, post-traumatic stress disorder, liver disease, autoimmune diseases, or severe psychiatric disorders (e.g., severe depression that resists treatment or impacts ability to function; schizophrenia). As can be seen in Table 3, the samples for all three groups were diverse in ethnicity, religious beliefs, socioeconomic status, level of education, and field of study. Table 4 shows baseline continuous demographic variables such as age, and the six outcome variables: perceived stress, positive emotions, daily spiritual experiences, heart rate, diastolic and systolic blood pressure.

**Table 3:** Demographics at baseline.

	Group								
	Tamarkoz		Stress management		Waitlist		Total		
	*N*	%	*N*	%	*N*	%	*N*	%	*P*
**Gender**									
Male	7	24.10	14	31.80	5	16.7	26	25.2	.334
Female	22	75.90	30	68.2	25	83.30	77	74.8	
**Religious preference**									
All Christian groups	3	10.30	17	38.60	7	24.10	27	26.50	.073
Other religions	9	31.00	10	22.70	5	17.20	24	23.50	
Atheist/agnostics/none	17	58.60	17	38.60	17	58.60	51	50.00	
**Ethnicity or race**									
Caucasian (other than Hispanic)	6	20.70	12	27.30	15	51.70	33	32.40	.146
Hispanic or Latino	4	13.80	8	18.20	4	13.80	16	15.70	
Black or African American	0	0.00	4	9.10	1	3.40	5	4.90	
Asian/Pacific Islander	13	44.80	13	29.50	6	20.70	32	31.40	
Other	6	20.70	7	15.90	3	10.30	16	15.70	
**Did either parent graduate from college?**									
No	5	17.20	9	20.50	7	24.10	21	20.60	.369
Yes, both parents	18	62.10	30	68.20	13	44.80	61	59.80	
Yes, mother only	4	13.80	4	9.10	6	20.70	14	13.70	
Yes, father only	1	3.40	1	2.30	3	10.30	5	4.90	
Don’t know	1	3.40	0	0.00	0	0.00	1	1.00	
**Highest education level completed by either parents (or person who raised participant)**									
Did not finish high school	1	12.50	5	62.50	2	25.00	8	100.00	.752
High school diploma or G.E.D	2	28.60	2	28.60	3	42.90	7	100.00	
Attended college but did not complete degree	3	60.00	0	0.00	2	40.00	5	100.00	
Associate's degree	2	33.30	3	50.00	1	16.70	6	100.00	
Bachelor's degree	9	36.00	9	36.00	7	28.00	25	100.00	
Master's degree	7	23.30	14	46.70	9	30.00	30	100.00	
Doctorate degree	5	25.00	10	50.00	5	25.00	20	100.00	
**University student classification**									
Freshman or first-year	8	27.60	16	36.40	10	34.50	34	33.30	.627
Sophomore	3	10.30	8	18.20	4	13.80	15	14.70	
Junior	10	34.50	6	13.60	7	24.10	23	22.50	
Senior	6	20.70	7	15.90	4	13.80	17	16.70	
Graduate student	2	6.90	7	15.90	4	13.80	13	12.70	
**Undergrad or graduate**									
Undergraduate	26	92.90	36	81.80	24	82.80	86	85.10	.400
Graduate	2	7.10	8	18.20	5	17.20	15	14.90	
**Marital status**									
Married	1	3.40	1	2.30	0	0.00	2	2.00	.627
Single	28	96.60	42	97.70	29	100.00	99	98.00	
**Major**									
Arts and humanities	3	10.30	5	11.40	4	13.30	12	11.70	.842
Biological sciences	3	10.30	8	18.20	2	6.70	13	12.60	
Physical sciences	6	20.70	11	25.00	7	23.30	24	23.30	
Social sciences	8	27.60	10	22.70	7	23.30	25	24.30	
Interdisciplinary studies and undeclared	6	20.70	4	9.10	6	20.00	16	15.50	
Business	3	10.30	6	13.60	3	10.00	12	11.70	
Missing information on major	0	0.00	0	0.00	1	3.30	1	1.00	
**Best estimate of family’s household income**									
$20,000–$40,000	11	37.90	9	20.50	4	14.30	24	23.80	.244
$50,000–$70,000	3	10.30	11	25.00	6	21.40	20	19.80	
$80,000–$90,000	2	6.90	7	15.90	6	21.40	15	14.90	
$100,000 or more	13	44.80	17	38.60	12	42.90	42	41.60

**Table 4:** Continuous demographic and baseline outcome variables.

	Groups																
	Tamarkoz				Stress management				Waitlist				Total				
	*N*	Mean	95% CI		*N*	Mean	95% CI		*N*	Mean	95% CI		*N*	Mean	95% CI		*P*
			Lower	Upper			Lower	Upper			Lower	Upper			Lower	Upper	
PSS^a^	29	19.45	16.87	22.03	44	18.80	16.86	20.73	30	18.97	16.72	21.22	103	19.03	17.79	20.27	.911
DPES^b^	29	162.97	151.31	174.62	44	158.91	150.07	167.75	29	163.83	154.86	172.79	102	161.46	155.98	166.94	.723
DSES^c^	29	35.38	28.12	42.64	44	35.43	29.53	41.33	29	28.07	23.12	33.01	102	33.32	29.81	36.83	.174
SBP	25	100.64	95.80	105.48	41	103.34	100.22	106.47	24	102.17	97.01	107.33	90	102.28	99.97	104.58	.630
DBP	25	66.84	63.59	70.09	41	69.68	67.23	72.13	24	71.58	68.78	74.39	90	69.40	67.80	71.00	.089
HR	25	71.12	67.04	75.20	41	76.20	72.66	79.73	24	73.79	69.71	77.87	90	74.14	71.93	76.36	.164
Age	29	20.34	19.32	21.37	44	20.18	19.26	21.10	29	20.45	19.39	21.51	102	20.30	19.75	20.86	.924
Grades	29	3.59	3.42	3.75	41	3.58	3.45	3.72	29	3.68	3.53	3.83	99	3.61	3.53	3.69	.583
HW	29	1.86	1.45	2.28	44	1.55	1.24	1.85	29	1.79	1.44	2.15	102	1.71	1.51	1.90	.368
EMW	29	121.81	88.69	154.93	44	131.99	100.09	163.89	30	104.58	67.16	142.00	103	121.14	101.88	140.40	.506

*DSES* Daily Spiritual Experiences Scale, *DPES* Dispositional Positive Emotions, *PSS* Perceived Stress Scale, *SBP* Systolic Blood Pressure, *DBP* Diastolic Blood Pressure, *HR* Heart Rate, *HW* Hours Worked, *EMW* Exercise minutes/week.

^a^Range was from 0 for lowest to 40 for highest stress.

^b^Range was from 38 for lowest to 266 for highest positive emotions.

^c^Range was from 16 for lowest to 94 for highest daily spiritual experiences.

### Recruitment

Recruitment began during the first week of the fall semester. Flyers were posted throughout the Berkley campus, and provided to the campus student health center for posting at their stress management resource center. The intervention group was recruited from the Tamarkoz class for only first-time Tamarkoz students. Participants were not provided extra credit for the study, and the instructor was not informed which of her students had enrolled in the study.

**Figure 1:**
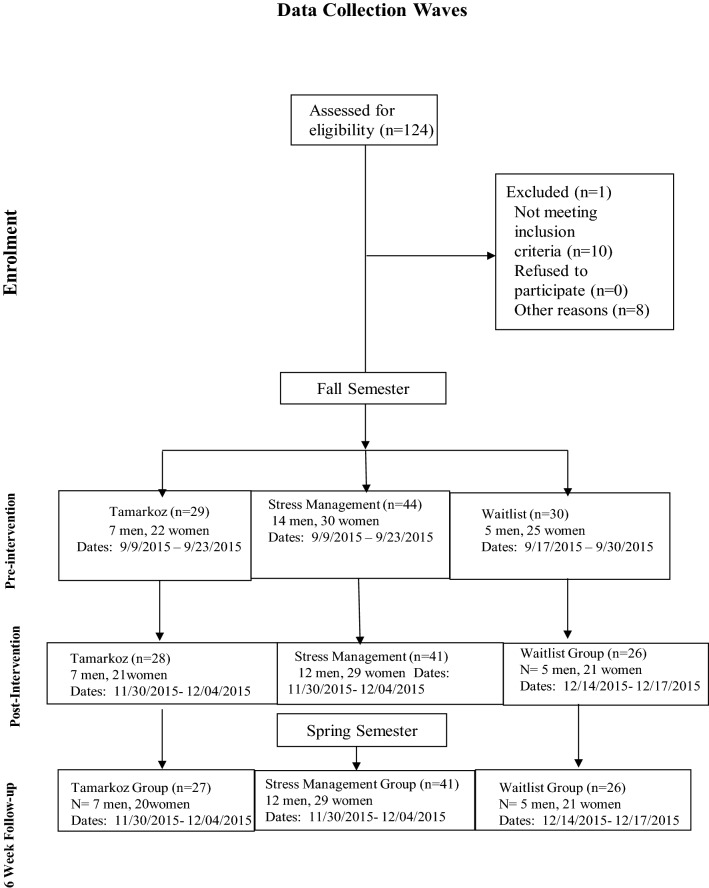
Recruitment and data collection.

The Waitlist control group was recruited from the waitlist of the Tamarkoz class and other students who wanted to enroll into the class. Participants in the waitlist group did not utilize stress management resources at the campus’ student health center for the duration of the study and received priority registration for Tamarkoz for the following semester. Students were informed that they would not be dropped from the study if they decide at any time to use the stress management resources. Each self-report questionnaire asked students whether they utilized the campus health center’s stress management resources. The participants in the Stress Management Resources (standard practice control) group utilized the campus health center's stress management resources on an as-needed basis. Their resources included access to health coaching, individual counseling and psychological services, group counseling, online educational information about self-care for stress management, pet hugs, and use of a massage chair. Baseline was completed prior to the intervention group's first exposure to Tamarkoz class. Figure 1 provides information on the number of participants and the dates for each data collection wave.

### Compensation

Once students contacted the researcher and self-reported meeting the eligibility criteria, they were provided an appointment to meet with the researcher on campus in a designated room where all participants gave their informed consent and had blood pressure and heart rate measurements taken. To the extent possible, participants of each group were met with separate appointments to reduce chances that group members would communicate with each other about the study. Participants were compensated up to $10 in Amazon gift cards for each of the following items if they were complete and provided on time: (a) blood pressure/heart rate measurements, and (b) the online self-report survey.

### Measures and covariates

The self-report questionnaires, blood pressure, and heart rate were assessed at baseline, end of course (12 weeks) and 18 weeks. The questionnaires were emailed as an online survey through Qualtrics to participants for their participation at every data collection time point. In order to confidentially track each participant over time, each person was associated with a specific participant code.

#### Perceived stress

Perceived stress was measured with the 10-item Perceived Stress Scale (PSS, α = 0.88 in our data)^74^. The scale measures the degree to which one perceives situations in one’s life as stressful, such as “In the last month, how often have you been able to control irritations in your life?” The scale includes six negatively worded items that represent stress and four positively stated items that represent confidence and control. Responses range from 0 = never to 4 = very often. Items were summed across the scale providing a range of 0 to 40. Higher scores on the scale indicate higher levels of perceived stress.

#### Dispositional positive emotions

Positive emotions were measured with the Dispositional Positive Emotions Scale (DPES)^75^. This scale is a 38-item (α = 0.94) self-report questionnaire. An example of an item from the scale is, “I see beauty all around me.” The responses range from 1 = strongly agree to 7 = strongly disagree with possible scores ranging of 38 to 266. Items were summed across the scale and then reversed so that higher scores indicate more positive emotions.

#### Daily spiritual experiences

Spirituality levels were measured with the Daily Spiritual Experience Scale (DSES)^76^. This scale consists of 16-items (α = 0.95) that assess an individual’s perception of the transcendent, divine, or holy in their daily life. The constructs of the scale represent common processes in religiousness and spirituality within daily life. An example of an item from the scale is, “I find strength in my religion or spirituality.” Reponses range from 1 = many times a day to 6 = never with scores ranging from 16 to 96. The 16th item on the scale is reversed coded and then the items were summed. The total score was reversed so that higher scores indicate more daily spiritual experiences.

#### Blood pressure and heart rate

Blood pressure and heart rate measurements were taken with an Omron 10 Plus Series Upper Arm Blood Pressure Monitor, which is a validated instrument approved and recommended by dabl Educational Trust for accuracy. Three measurements were taken with a thirty second pause between, then these three were averaged.

#### Control variables

Gender, age, ethnicity, religious preference, and self-reported exercise minutes per week at baseline were controlled in this analysis. Because there were fewer than ten people in many of the religious preferences, people were dichotomized into Christians, other religion or atheists/agonistics. The Tamarkoz group had a writing space on their survey where they had the option to share about their experience with Tamarkoz.

### Experimental intervention

The Tamarkoz group participants had one lecture and one meditation practice per week. All Tamarkoz study participants were taught by the same instructor. The lecture portion of the class provides context for the Tamarkoz practice and as stated on the course syllabus provides “Understanding of the principles, basic beliefs, and goals of Sufism, and the role Tamarkoz plays in achieving the aim of practical Sufism, which is self-knowledge.” According to the course syllabus, the class is based on the teachings of the Sufi Masters of the Maktab Tarighat Oveyssi (M.T.O.) Shahmaghsoudi, School of Islamic Sufism with particular focus on the teachings of Professor Angha. The goal of Sufism is self-knowledge; thus, the teachings presented in class elaborate on the elements of this goal. The Tamarkoz practice sessions include the following elements: Light stretches to release tension from the physical body. Then mind relaxation, and deep breathing exercises, followed by Movazeneh, and then deep relaxation, visualization, and then heart concentration, which focuses on the heartbeat. For more details, please see http://mtoshahmaghsoudi.org.

### Human subjects

Loma Linda University’s Institutional Review Board reviewed and approved all aspects of the research study (LLU-2016-5150225). All aspects of the study were performed in accordance with relevant guidelines and regulations, including informed consent. All students were eligible to register for the Tamarkoz course until capacity was met and all students were eligible to use the self-care stress-management resources at the campus student health center.

### Data analysis

There was only one independent variable: whether the participant was in the Tamarkoz group, the stress management control group, or the Tamarkoz waitlist control group. Six outcome variables were tested: perceived stress, positive emotions, daily spiritual experiences, systolic blood pressure, diastolic blood pressure, and heart rate. Participants with missing information on perceived stress, dispositional positive emotions, and daily spiritual experiences were excluded from the analyses resulting in a baseline sample size of 103 participants. Data for blood pressure and heart rate measurements were entered into SPSS using double entry to ensure accuracy. SPSS-23 was used for all statistical analyses. Baseline differences on categorical variables were tested using the χ^2^ goodness-of-fit test and on continuous variables using one-way ANOVA. For analyses that involved examining participants across time, the generalized linear mixed model was used. Linear mixed models, when used for analysis of repeated measures data, have advantages over the standard linear models approach; in particular, they enable us to deal effectively with both within- and between-person variability. This is because, among other things, they do not require the assumption of independence of observations over time^77^ and differences between groups can be modeled as random effects. Each of the six dependent variables had three effects tested: (a) wave, which tested whether there were consistent differences in a variable across time regardless of group; (b) group, which tested whether the average value for each group differed regardless of time; and (c) the group by wave interaction which tested whether the pattern of change over time differed from one group to another. For testing these three effects on the six outcome variables we used five variables as statistical controls: gender, age, ethnicity, religious preference, and baseline level of exercise. While none of these variables were significantly different at baseline, we decided that it would make sense to control for basic demographics and one health habit, exercise, that could have significant effects on stress management. The intent was to increase our power by controlling for extraneous variation.

## Results

### Baseline differences in outcome variables and comparison with scale norms

Table 4 shows that at baseline there were no statistically significant differences in means among groups in perceived stress, dispositional positive emotions, daily spiritual experiences, systolic and diastolic blood pressure and heart rate. Cohen's normative group for perceived stress had the following parameters: M = 16.78, SD = 6.86, 95% CI: [15.88, 17.68], n = 223. The results for perceived stress measurements for our groups were the following: Tamarkoz group: (t(250) = 1.38, *p* = 0.168); Stress Management group: (t(265) = 1.78, *p* = 0.077); Waitlist group: (t(251) = 1.63, *p* = 0.105). While our groups have higher means than the normative table, the differences are not statistically significant.

Differences between reported positive emotions in the normative table, whose participants were also students from a large West Coast University, and the groups in this study were statistically significant with the same age group and the following parameters: M = 4.98, SD = 0.71, 95% CI: [4.85, 5.11], n = 119, compared to baseline of the Tamarkoz group: (t(146) = 4.58, *p* < 0.001); the Stress Management group: (t(161) = 6.25, *p* < 0.001); and the Waitlist group: (t(146) = 4.66, *p* < 0.001).

The normative table for daily spiritual experiences was derived from the General Social Survey^78^, which was based on responses from the 16-item Daily Spiritual Experiences Scale taken in 2004 by people who were between the age group of 18–30 years old. These values were M = 47.87, SD = 18.00, 95% CI [45.73, 50.01], n = 272. At baseline, the normative numbers are different than our groups, and had statistically significantly higher daily spiritual experiences than the Tamarkoz group: (t(299) = 3.53, *p* = 0.001); the Stress Management group: (t(19.40) = 4.21, *p* < 0.001); and the Waitlist group: (t(13.00) = 5.76, *p* < 0.001).

#### Group differences in outcome variables

**Figure 2:**
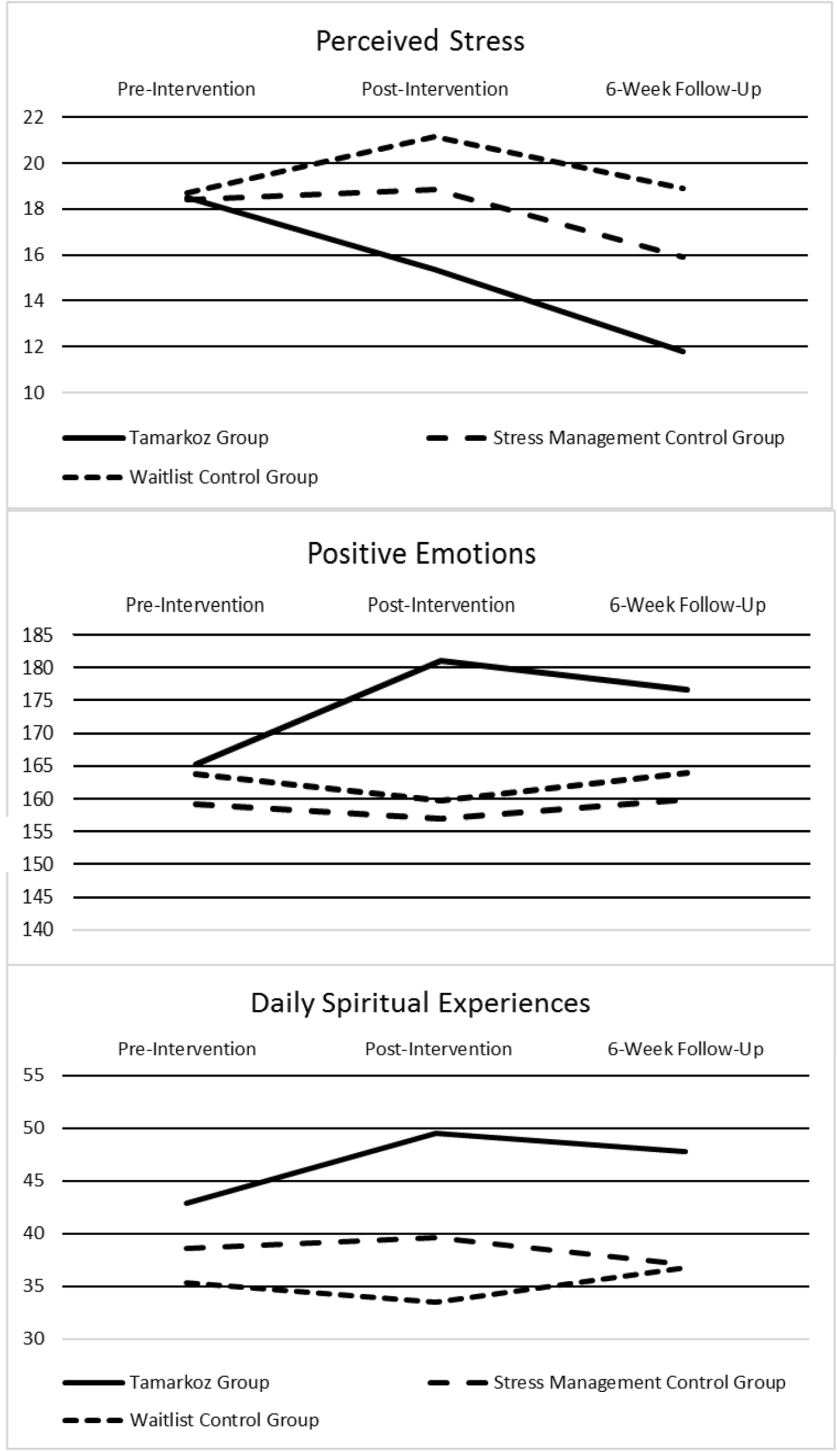
Outcomes variables of perceived stress, dispositional positive emotions and daily spiritual experiences using generalized linear mixed modeling with controls for gender, age, ethnicity/race, religious preference, and exercise minutes per week.

Table 5 shows results from the repeated measures analysis for all dependent variables while Table 6 shows the means and 95% confidence intervals for these same variables for each group at each of the three-time points. The means are adjusted for gender, age, ethnicity/race, religious preference, and exercise minutes per week. Figure 2 shows the patterns of these changes over time. There was significant difference in the use of stress management resources between the Stress Management group and the Tamarkoz group, such that the Stress Management group utilized them more.

**Table 5:** Repeated measures analysis using generalized linear mixed model for the six outcome variables with controls for gender, age, ethnicity/race, religious preference, and baseline exercise.

Outcome variable	Effect	F	df1	df2	p
**Perceived stress**					
	Wave^a^	11.460	2	273	.000
	Group^b^	4.311	2	273	.014
	Group × wave	4.311	4	273	.002
**Positive emotions**					
	Wave	1.737	2	273	.055
	Group	2.939	2	273	.178
	Group × wave	3.950	4	273	.004
**Daily spiritual experiences**					
	Wave	1.421	2	273	.243
	Group	3.609	2	273	.028
	Group × wave	3.878	4	273	.004
**Systolic blood pressure**					
	Wave	9.925	2	231	.000
	Group	1.640	2	231	.196
	Group × wave	1.088	4	231	.363
**Diastolic blood pressure**					
	Wave	12.368	2	231	.000
	Group	1.490	2	231	.228
	Group × wave	0.620	4	231	.649
**Heart rate**					
	Wave	2.218	2	231	.111
	Group	4.883	2	231	.008
	Group × wave	3.074	4	231	.017

^a^Wave was baseline, 12 weeks after baseline, and 18 weeks after baseline.

^b^Group was Tamarkoz treatment, stress management control, or Tamarkoz waitlist group.

**Table 6:** Means [95% CI] for dependent variables adjusted for gender, age, ethnicity/race, religious preference, and exercise minutes per week.

	Pre-intervention	Post-intervention	6-Week follow-up
**Perceived stress**^**a**^			
Tamarkoz group	18.50	15.39	11.77
	[16.00, 21.01]	[12.13, 18.65]	[8.95, 14.59]
Stress management control	18.42	18.87	15.93
	[16.66, 20.18]	[16.97, 20.77]	[13.89, 17.97]
Waitlist control group	18.69	21.17	18.92
	[16.38, 21.00]	[18.71, 23.62]	[16.09, 21.75]
**Dispositional positive emotions**^**b**^			
Tamarkoz group	165.26	181.10	176.65
	[151.74, 178.78]	[167.15, 195.06]	[162.87, 190.43]
Stress management control	159.10	157.00	159.90
	[149.49, 168.72]	[145.94, 168.06]	[148.86, 170.95]
Waitlist control group	163.81	159.78	164.00
	[153.30, 174.31]	[148.59, 170.97]	[151.64, 176.35]
**Daily spiritual experiences**^**c**^			
Tamarkoz group	42.84	49.51	47.74
	[35.87, 49.81]	[42.56, 56.46]	[39.65, 55.82]
Stress management control	38.55	39.63	37.10
	[33.73, 43.38]	[ 33.85, 45.41]	[32.50, 41.70]
Waitlist control group	35.28	33.50	36.71
	[29.84, 40.72]	[27.72, 39.28]	[30.92, 42.51]
**Systolic blood pressure**			
Tamarkoz group	104.87	108.21	106.05
	[100.65, 109.09]	[104.01, 112.41]	[102.25, 109.86]
Stress management control	106.18	111.82	106.52
	[103.54,108.81]	[108.69, 114.95]	[103.82, 109.23]
Waitlist control group	107.00	114.37	109.90
	[103.02, 111.00]	[108.96, 119.78]	[104.71, 115.10]
**Diastolic blood pressure**			
Tamarkoz group	67.65	73.33	71.90
	[64.40, 70.89]	[70.34, 76.32]	[69.35, 74.40]
Stress management control	70.37	75.26	72.27
	[68.04, 72.70]	[73.33, 77.19]	[69.78, 74.87]
Waitlist control group	72.41	75.10	74.69
	[69.57, 75.25]	[70.64, 79.56]	[70.54, 78.84]
**Heart rate**			
Tamarkoz group	72.76	69.77	76.07
	[68.64, 76.88]	[64.85, 74.69]	[71.00, 81.13]
Stress management control	77.92	82.24	79.51
	[74.62, 81.21]	[78.29, 86.20]	[75.29, 83.74]
Waitlist control group	75.92	81.71	79.44
	[72.03, 79.82]	[74.30, 89.12]	[72.21, 86.66]

^a^Maximum possible = 40, Minimum possible = 0.

^b^Maximum possible = 266, Minimum possible = 38.

^c^Maximum possible = 94, Minimum possible = 16.

#### Perceived stress

As shown in Table 5, the hypothesis that perceived stress would be lower for the Tamarkoz group was supported, with a significant group-by-wave interaction. Figure 2 demonstrates  that the Tamarkoz group showed a steady decrease in perceived stress over time, whereas the other two groups increased before dropping back toward baseline. At the second wave the sequential-Sidak, paired-comparison test showed that the Tamarkoz group’s stress was lower than both control groups, but the difference was significant only for the Waitlist group (t(273) = 2.98, *p* = 0.009). At the third wave, however, (6-week posttest) the Tamarkoz group was significantly different from both the Waitlist group (t(273) = 3.79, *p* = 0.001) and the Stress Management group (t(273) = 2.50, *p* = 0.026).

#### Dispositional positive emotions

This study also supports the hypothesis that Tamarkoz increases positive emotions in university students compared to a group utilizing standard stress management resources and a waitlist control group. Table 5 indicates a significant group-by-wave interaction. Figure 2 and Table 6 show that dispositional positive emotions increase following the intervention in the Tamarkoz group but stay about the same in the two control groups. The sequential-Sidak, paired-comparison tests demonstrate that at the second wave, positive emotions for the Tamarkoz group are significantly higher than the Stress Management group (t(273) = 3.30, *p* = 0.003) and the Waitlist group (t(273) = 2.89, *p* = 0.008).

#### Daily spiritual experiences

This study supports the hypothesis that Tamarkoz increases daily spiritual experiences. Over half of the participants in the Tamarkoz group were atheists, agnostics, or had no religious preference yet increases in daily spiritual experiences were still observed, demonstrating that the technique is not limited to those who self-designate as religious or who declare a religious affiliation. Table 5 demonstrates a significant group-by-wave interaction in daily spiritual experiences. The pattern denoted in Fig. 2 illustrates the increase in daily spiritual experiences for the Tamarkoz group relative to the other two groups in wave 2 and 3. Sequential-Sidak, paired-comparison tests at the second wave demonstrate that the Tamarkoz group has statistically significant higher daily spiritual experiences than the Waitlist group (t(273) = 3.60, *p* = 0.001), and marginally significant compared with the Stress Management group (t(273) = 2.24, *p* = 0.051). At the third wave, the Tamarkoz group had higher daily spiritual experiences than the Stress Management group (t(273) = 2.34, *p* = 0.059) and the Waitlist group (t(273) = 2.26, *p* = 0.059), but these differences are only marginally significant.

#### Systolic and diastolic blood pressure and heart rate

**Figure 3:**
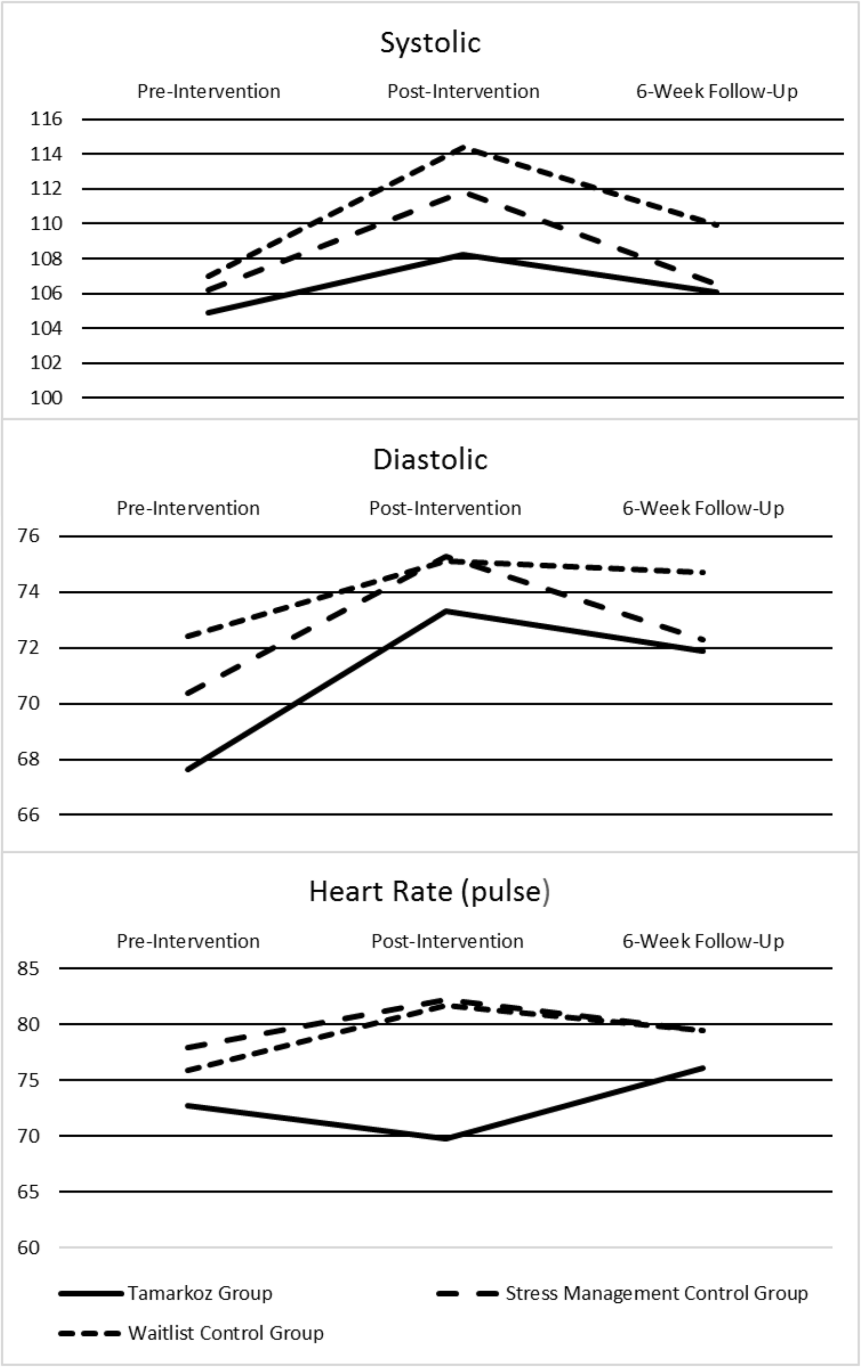
Outcomes variables of systolic blood pressure, diastolic blood pressure and heart rate using generalized linear mixed modeling with controls for gender, age, ethnicity/race, religious preference, and exercise minutes per week.

Figure 3 illustrates that the Tamarkoz group had lower systolic and diastolic blood pressure at the second wave and the third wave compared to the Stress Management group and the Waitlist group, however the differences were not statistically significant.

Self-report of reduced perceived stress in the Tamarkoz group was supported by significantly reduced heart rate. Sequential-Sidak, paired-comparison tests indicate a significant group-by-wave interaction for heart rate such that the Tamarkoz group had significantly lower heart rate than the Stress Management group (t(231) = 4.09, *p* < 0.001) and the Waitlist group (t(231) = 2.82, *p* = 0.011) at the second measurement wave. Figure 3 also shows that the Tamarkoz group had reduced heart rate during the second wave and continued to have lower heart rate than the two other groups during the third wave.

## Discussion

University students' stress, depression and anxiety are neglected public health issues which have significantly negative impacts on students’ quality of life, academic performance, and prospective occupational success. Health-related habits formed during the period of university and college life may be difficult to change later on^13^. Research studies on health and wellness emphasize the need for multidimensional, whole person wellness programs that include physical, emotional, behavioral, and spiritual elements^46^.

Studies show that college and university students with high levels of distress tend to use more substances such as alcohol and illicit drugs and this creates a real public health challenge for American college campuses^8,79^. Challenges can be exacerbated and made more difficult for college counselors to address because of the complex ways in which these factors are related to personal characteristics and contemporary culture^15^. Given the rather consistently stressful college environment, the diversity-related challenges, and the financial costs associated with more traditional medical treatments, it is important to find innovative approaches for addressing the stress-related health issues. There is a good deal of evidence for the benefits of meditation on various aspects of health. This study adds to the literature by demonstrating that during the most stressful time of the semester, compared to two control groups, the participants in the Tamarkoz group experienced significantly more positive emotions, more daily spiritual experiences, reduced perceptions of stress, and reduced heart rate, which suggests the body’s effective regulation of cardiovascular reactivity.

The outcomes of this study are consistent with the idea that positive emotions may effectively counteract negative emotions and thereby improve psychological resiliency and speed recovery from cardiovascular events induced by negative emotions^80^. The broaden-and-build theory^80^ of positive emotions posits that they prepare the individual to better deal with stressful events and, hence, lessen perceived stress and enhance emotional well-being.

Additionally, our results showed a significant relationship between positive emotions and daily spiritual experiences. It has been suggested that spiritual people tend to experience more positive emotions, such as feelings of fulfillment, which may provide an inner strength that protects against negative feelings of anxiety or despair^81^.

Increase in spirituality and practices such as meditation and prayer elicit relaxing physiological effects on the body that improves health outcomes^44,52,82^ and may thereby decrease stress. Spirituality can positively influence psychological well-being, as it can buffer against adverse stressors of daily life and moderates the effects of stress even without religious affiliation^81^. A fair number of participants (approximately 57%) in the Tamarkoz group were atheists, or agnostics. The results provide important implications for health promotion and wellness programs in that Tamarkoz increases daily spiritual experiences regardless of religious affiliation.

Tamarkoz techniques provides students with tools to manage stress, regulate their emotions and increase spiritual experiential details in daily life. It can be practiced at any time, and in any location preferred by the participant. It does not require a gym, or a special meditation location, and does not require hours of commitment in a day. This is especially important in that students are taught techniques to use on their own, which may lead fewer students needing to utilize support services.

Limitations of the study included that the sample sizes of the groups were not large, and thus it is possible that there was insufficient power to pick up the association in the posttests for all three groups in some variables. Furthermore, the Stress Management group (as a comparison group), was not required to engage in a specific number of hours of exposure to a stress management resource. However, one purpose of this study was to determine whether the stress management resources available to use independently was sufficient. Additionally, because there were no statistically significant differences on demographics, perceived stress, positive emotions, or daily spiritual experiences at baseline for the three groups, we did not control for previous meditation practice. It may be, however, that those with prior experience with other meditation modalities might be able to transition into a meditative practice more quickly. Therefore, future studies with larger sample sizes might benefit from including this as a control.

Strengths of this study were use of validated scales and objective, physiological measures with clinically validated monitors, and comparison groups, which allowed to control for a variety of variables. The Stress Management group may control for possible placebo effects as those utilizing the stress management program would believe, as would the Tamarkoz group, that they were receiving something to help with stress, yet the Tamarkoz group was more positively affected. The Waitlist Control group was recruited from the waitlist of the Tamarkoz group, which served as an appropriate comparison group as participants had the same interests in Tamarkoz.

## Conclusion

The study demonstrates the health promotion benefits of incorporating a specific type of meditation class as a curriculum for university students. Based on the study results, it seems that just providing resources for stress management on campus is not enough, but rather providing a course that incorporates spirituality and teaches skills as well as the opportunity to practice and develop these skills, will not only reduce perceived stress, but also increase positive emotions and daily spiritual experiences. In a national survey of United States college students, over 80% indicated interest in spirituality, however inner development of students, such as spirituality, emotional maturity and self-understanding has been neglected at higher education institutions^83^. Thus, universities may need to offer courses on learning techniques that will counter stress, while fostering spirituality—techniques such as those offered in Tamarkoz training— particularly since it seems to show some advantage over the usual stress management resources offered by a student health center. It is interesting that in the two control groups of this study, the stress went up at the time of finals, which suggests there are in fact problems on college campuses that need to be dealt with in some way. One of these ways may be a course such as Tamarkoz.

A future study could require participants in a stress management group to utilize stress management resources for the same duration as the Tamarkoz group. Another study could explore whether the decrease stress in university students who participate in Tamarkoz would potentially decrease symptoms of anxiety or depression. A suggestion for the next study is to incorporate random assignment to conditions.

## Data Availability

The dataset generated and analyzed during the current study are available in the American Educational Research Association Data Repository, https://doi.org/10.3886/E137281V1.
